# Accurate and Scalable Techniques for the Complex/Pathway Membership Problem in Protein Networks

**DOI:** 10.1155/2009/787128

**Published:** 2010-02-23

**Authors:** Orhan Çamoğlu, Tolga Can, Ambuj K. Singh

**Affiliations:** ^1^Department of Computer Science, University of California, Santa Barbara, CA 93106, USA; ^2^Department of Computer Engineering, Middle East Technical University, 06531 Ankara, Turkey

## Abstract

A protein network shows physical interactions as well as functional associations. An important usage of such networks is to discover unknown members of partially known complexes and pathways. A number of methods exist for such analyses, and they can be divided into two main categories based on their treatment of highly connected proteins. In this paper, we show that methods that are not affected by the degree (number of linkages) of a protein give more accurate predictions for certain complexes and pathways. We propose a network flow-based technique to compute the association probability of a pair of proteins. We extend the proposed technique using hierarchical clustering in order to scale well with the size of proteome. We also show that top-k queries are not suitable for a large number of cases, and threshold queries are more meaningful in these cases. Network flow technique with clustering is able to optimize meaningful threshold queries and answer them with high efficiency compared to a similar method that uses Monte Carlo simulation.

## 1. Introduction

Deciphering the complex networked organization of proteins is essential to understand the functions of life. A protein network shows physical interactions as well as functional associations like inhibition, activation, and phosphorylation between the proteins of an organism. Such networks are being constructed for different species. A biologically motivated problem is to predict new members of a partially known protein complex or pathway of an organism. In this problem, a particular core set of proteins is known, but the biologists are not confident that this core set is complete. The goal is to find a list of candidate proteins, preferably with an associated probability of membership in the partially known complex or pathway.

Given a protein-protein interaction network, various computational techniques exist to solve the complex membership problem. They can be classified into two categories as methods that normalize edge weights incident on a node (random walks [1] and diffusion kernels [2]), and methods that do not normalize edge weights (Markov Random Field [3] and network reliability by Monte Carlo simulation [4]). Below, we give a brief overview of these methods.

Random walk method [1] simulates a random walker that starts at a source node and visits other nodes through connecting edges. The probability of finding the random walker at a certain node gives the affinity of that node to the starting node. The details of the random walk method can be found in Lovasz [1]. Similar in principle, diffusion kernels provide a global similarity metric for a graph. The computation of a diffusion kernel is based on the Gaussian radial basis function kernel [2].

Markov Random Field (MRF) method is based on belief propagation. Letovsky and Kasif [3] used MRF for analyzing protein networks for function assignment. However, a negative labeled set is needed to prevent propagation of positive labels to the whole network. Nabieva et al. [5] also uses a network flow-based technique for whole-proteome prediction of protein function. Two-terminal network reliability is another technique that can be used to discover close proximity proteins in a protein network. The exact solution to the network reliability problem is NP-hard [6]. Monte Carlo simulation (referred to as MCS throughout the paper) provides an approximation to this problem [4]. MCS, similar to MRF method, does not penalize the incoming edges of a node when computing the *connectivity* of a protein to the core complex/pathway. However, this approach is computationally intensive for large networks. Therefore, more efficient techniques that can scale well with the network size are needed.

In this paper we propose a new technique, *Net-Flow*, that is based on network flow for the complex/pathway membership problem in protein interaction networks. This technique is able to compute theoretically proven bounds on the * reliability* of candidate proteins. Here, we define * reliability of a candidate protein* as the probability that there is a path of interacting proteins between the candidate protein and the query complex/pathway. We improve Net-Flow technique further by integrating a clustering component. We consider *threshold queries* that return candidate proteins satisfying a threshold probability of membership in the query complex/pathway. We show that Net-Flow produces optimum results with high efficiency for threshold queries.

In the recent years, studies on analysis of protein networks mostly focus on integrating various sources of information for identification of novel functional modules [7–9] and understanding network evolution to identify conserved modules in multiple species [10–12]. In this paper, we focus on the specific problem of finding new members of a partially known complex/pathway of a single species. We assume that no further information apart from the interaction of proteins is known. Our technique does not assume conservation of functional modules therefore is able to handle species specific complexes and pathways.

In Section 2 we discuss the difference between the two classes of analysis techniques. We propose the network flow technique in Section 3 and discuss the integration of clustering in Section 4. We present our experimental results in Section 5. We conclude with a brief discussion.

## 2. The Difference of High Degree Nodes

Two groups of network analysis methods treat high degree nodes on the network differently. The methods that normalize the sum of weights of the number of the outgoing edges for each node implicitly assume that high degree nodes are more likely to interact with a random protein and functional interactions involving a high degree node are less important than the functional interactions involving nonhigh degree nodes. The methods that do not use the aforementioned normalization give more importance to the weights of each edge, thus the experimental evidence for inferred interaction is used.

**Figure 1:**
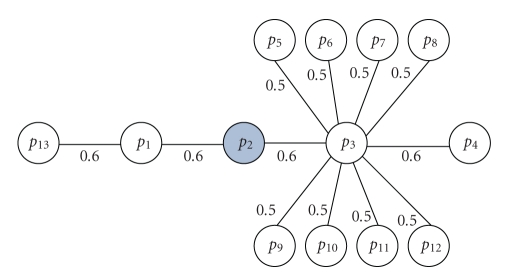
A probabilistic network that represents the functional interactions between thirteen proteins. Proteins *p*_13_, *p*_1_, *p*_2_, *p*_3_, and *p*_4_ are members of a pathway and *p*_3_ is a multifunctional protein associated with multiple proteins (high degree node).

The effect of normalizing incoming edges is illustrated in the following example. Suppose that a probabilistic network given in Figure 1 is used to model the interactions between thirteen gene products. Given that protein *p*_2_ is part of a pathway, we want to rank all the remaining proteins in the network based on their probability of being a member of the same pathway.

 Based on the connections in the network, it is reasonable to assume that *p*_1_ and *p*_3_ have equal probabilities of participating in the pathway of interest since they are both connected to the *p*_2_ with equal weights. However, the random walk and diffusion kernel methods, which normalize the edges incident on a node, assign a larger probability to *p*_3_ than *p*_1_. The random walk method with a restart probability of 0.5 gives an affinity of 0.176 to *p*_3_ and an affinity of 0.157 to *p*_1_. As the restart probability decreases, node *p*_3_ gets higher scores, and as the restart probability increases and approaches to 1 (more local structure around *p*_2_), score of *p*_3_ will converge to the score of *p*_1_. On the other hand, a network reliability technique that does not normalize based on the edges incident to a node always gives equal probabilities (0.6) to *p*_1_ and *p*_3_. A more interesting pair of proteins to investigate is the pair *p*_13_ and *p*_4_. Because of the high degree node between the source node *p*_2_ and *p*_4_, *p*_4_ always gets a probability (using random walks) that is lower than *p*_13_. For restart probability of 0.5, *p*_13_ receives an affinity of 0.039, whereas *p*_4_ receives 0.010. For MCS, both nodes receive equal values of 0.6 · 0.6 = 0.36. This example clearly demonstrates the difference between the two groups of analysis methods.

**Figure 2:**
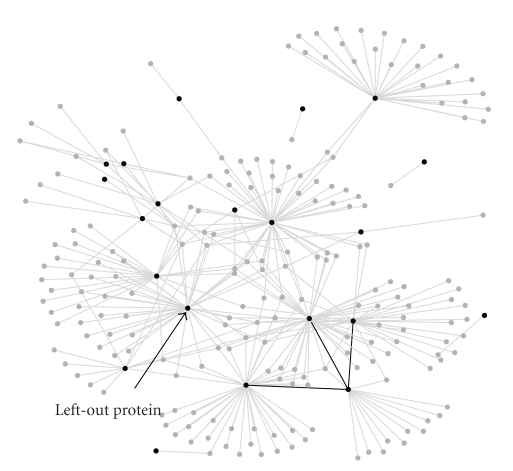
The subnetwork of *pyruvate metabolism* pathway of Saccharomyces cerevisiae based on ProNet [4]. The members of the pathway are plotted with black points and the connecting proteins are plotted with gray nodes. The edges between the complex members are indicated bold lines.

Cases similar to the one discussed above exist regularly in protein interaction networks since they have been shown to be scale-free networks [13] meaning that there are a number of proteins that are connected to many proteins. The *pyruvate metabolism* pathway of Saccharomyces cerevisiae, yeast, from KEGG database [14] is an example of this case. The subnetwork based on ProNet [4] for this pathway is depicted in Figure 2. In the figure, members of the pathway are plotted with black nodes, and neighboring proteins are plotted with gray nodes. Edges that connect members of the pathway are marked with bold lines. A membership query is constructed by using three strongly connected members, and the rank of the left-out protein, YOR374w (Mitochondrial aldehyde dehydrogenase) is computed. Since most of the proteins in the pathway are connected to a large number proteins, we expect a significant difference in rankings of methods that penalize the high degree nodes and the ones that do not. A random walk technique that penalizes high degree nodes computes the rank of left-out protein as 217, where the network reliability based technique (MCS) computes the rank as 72. This significant difference supports that the methods that do not penalize high degree nodes have advantages over the ones that do penalize. Note that, there may be certain subgraphs in which methods that normalize edge weights produce biologically more acceptable reliability values. However, with the synthetic and real examples given above, we demonstrate that normalizing edge weights may lead to inaccurate associations when the local neighborhood of a partially known complex/pathway adopts a scale-free topology.

MCS technique, which does not penalize high degree nodes, can be used to answer pathway membership queries. It provides a good approximation to network reliability problem. However it has a number of disadvantages. First of all, the approximations computed by MCS do not provide any proven bounds. To ensure the correctness of the approximation a high number of samples have to be used. Also this approach is not scalable for large networks. The number of samples used increases with the size of the network to ensure the correctness of the approximation. Thus for large networks this approach faces longer running times. Another disadvantage of the MCS is that it bounds the length of paths between functionally related proteins, a cutoff of length of 4 is used by Asthana et al. [4]. This approach clearly fails for protein pairs for which strong evidence of interaction is yet to be discovered.

## 3. Net-Flow Technique

As we have shown in the previous section, methods that do not penalize the high-degreenodes have advantages over the ones that penalize the high-degreenodes. However current approaches like MCS have some shortcomings. We propose to solve this shortcoming by borrowing ideas from network analysis and linear programming to develop a new algorithm that gives accurate results in a scalable manner.

Two-terminal network reliability [15] in communication networks is a quantitative measure of reliability of links between two nodes. Much like protein interaction networks, there is a weighted edge between two nodes if there is a communication link between these nodes. The weight on the edge represents the probability that the link is operational at any given time. *Two-terminal network reliability* is, then, defined as the probability that there is an operational path between two given terminals. In protein interaction networks, we can define the two-terminal reliability as the probability that there is a path of interacting proteins between two given proteins. This reliability can also be viewed as the probability that at least one of paths between two nodes is functional, or, 1—probability that all paths fail.

To compute two-terminal reliability, one has to compute all the paths between two proteins and compute reliability of each path separately. The computation of the cumulative reliability is more complicated, since the effect of the edges shared on separate paths has to be considered. To overcome this complication, we can use edge disjoint paths and compute a lower bound on the reliability. Assume that we wish to compute the reliability between *s* and *t*, and there are *d* edge disjoint paths between *s* and *t*. The probability that all paths fail is ∏ _*i*=1_^*d*^ *P*_*i*_, where *P*_*i*_ represents the failure probability of the *i*th path. Given *l*_*i*_ as the length of the *i*th path, *P*_*i*_ is computed by *P*_*i*_ = 1 − ∏ _*j*=0_^*l*_*i*_^ *r*_*j*_, where *r*_*j*_ is reliability of the *j*th link on the path. The lower bound of reliability, *R*, between *s* and *t* is the probability that at least one of the paths is operational

(1) $$\begin{array}{c} R\geq 1-\overset{d}{\underset{i=1}{\prod }}P_{i}. \end{array}$$

**Figure 3:**
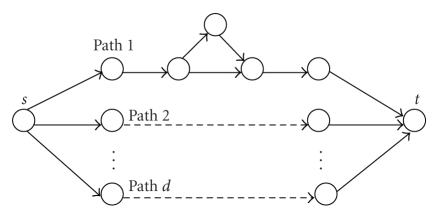
There are *d* edge disjoint paths between *s* and *t*. The first disjoint path shows two overlapping paths that share the first two and the last two edges on the path. Only one of these paths is used in reliability computation, that is, the one which maximizes flow. The reliability between *s* and *t* depends on the probability of at least one of these edge-disjoint paths being operational.

Edge disjoint paths between *s* and *t* are illustrated in Figure 3. Note that the bound computed by (1) is a theoretically proven bound unlike the other methods like random walks.

The computation of bounds on reliability is relatively easy if the edge disjoint paths are known. These paths can be computed by a simple breadth first search or via more complicated maximum flow techniques. The best technique would be the one that computes the maximum number of edge-disjoint paths since the bound in (1) gets tighter as the number of paths increases. As stated in *Menger's Theorem* [16], the maximum number of edge disjoint paths in a graph is equal to the minimum number of edges in an *s* − *t* cut. The minimum cardinality *s* − *t* cut can be computed by the maximum flow between *s* and *t*, where the capacity of each edge is set to 1.

For analysis of protein interaction networks, we need to compute reliability between a set of nodes that represent the member proteins of a query (i.e., a pathway or a complex) and the remaining proteins in the network. Each protein in the network becomes the sink in turn, while the member proteins represent the source nodes. The reliability of a sink node is then defined as the probability that there is a path between the sink node and one of the source nodes. The extension of a single source node to multiple source nodes can be easily accomplished by setting positive supply values for the sources nodes. Capacity of each edge is set to 1 and the cost of the edges are equal to link reliability, the probability that two proteins interact. We then compute the maximum flow between the sources and the sink by using linear programming. We impose flow constraints on the nodes as well as capacity constraints on the edges, and feed the problem to CPLEX, a linear programming solver [17], for linear programming solution of the maximum flow. Edge disjoint paths are constructed by using edges with flow in the maximum flow solution. These edge disjoint paths are considered to compute bounds on reliability by using (1). The overall algorithm for network flow technique is summarized in Algorithm 1. 

## 4. Addressing Scalability via HierarchicalClustering

Although our proposed solution to two-terminal network reliability problem can provide proven and tight bounds, it suffers from running time performance similar to MCS. A way to scale it is by addressing the two bottleneck steps in Algorithm 1: step 2 and step 4. To compute nodes with the best reliability with respect to the query proteins, a computation-intensive linear programming instance (step 4) is run a large number of times (step 2). So the algorithm can be scaled by either reducing the running time of an instance or the number of instances to run.

**Figure 4:**
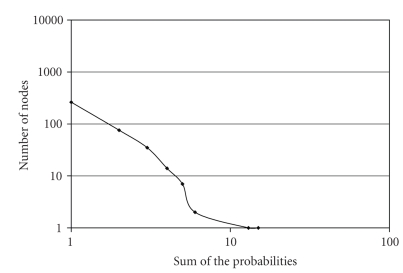
Histogram of sum of outgoing edges probabilities of nodes. On the yeast network, the weights of the outgoing edges of a node are summed and the number of nodes with the same weight is plotted. The curve follows the power law, which is essential to scale-free graphs.

These two points can be addressed by a clustering approach. Since protein interaction networks have been identified as scale-free graphs because a high number of nodes have low degrees and a small number of nodes have high degrees [13], they respond well to clustering. For the yeast network [4] the histogram of the sum of the probabilities of the outgoing edges of a node is plotted in Figure 4 to support this claim. Clustering of the networks is used to compute bounds for the proteins in the clusters and these bounds are used to speedup both of the bottleneck steps. Proteins that are most likely to interact with each other are clustered into the same cluster, and the interaction probability of proteins from different clusters is minimized. Then the bounds on the maximum and minimum reliabilities of a protein in each cluster are computed.

For the computation of the maximum bounds, we create a cluster graph. The cluster graph of an interaction network, *G* = (*V*, *E*), is defined as *G*′ = (*V*′, *E*′), where the vertices are clusters, and for *v*_*i*_′, *v*_*j*_′ ∈ *V*′, (*v*_*i*_′, *v*_*j*_′) ∈ *E*′ if and only if ∃*p*_*i*_, *p*_*j*_ ∈ *V* such that *p*_*i*_ ∈ *v*_*i*_′, *p*_*j*_ ∈ *v*_*j*_′ and (*p*_*i*_, *p*_*j*_) ∈ *E*. The weight of an edge (*v*_*i*_′, *v*_*j*_′) in the cluster graph is defined as max (weight(*p*_*i*_, *p*_*j*_)) for all *p*_*i*_ ∈ *v*_*i*_′, *p*_*j*_ ∈ *v*_*j*_′. So, in a cluster graph there is an edge between two clusters if a pair of proteins in these clusters have probability of interaction and the weight of the edge is the maximum probability of such pairs.

**Figure 5:**
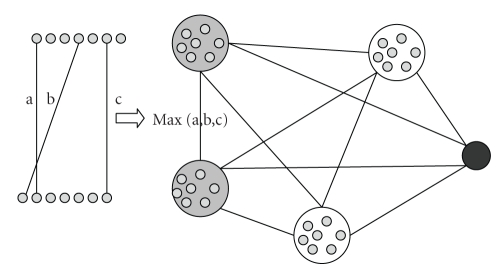
Construction of a cluster graph. Four clusters and a source node, the black circle, are shown. The weight of the edge between two gray clusters is computed by the maximum weight edge between their members in the original network (shown on the left). In the original network, there are three edges with weights a, b, and c between the seven nodes in each cluster. In the cluster network, an edge is put between these two clusters because there exists at least one pair of proteins in separate clusters that interact. The weight of this edge is the maximum of a, b, and c.

After the construction of the cluster graph as described, it is used in the flow algorithm. The query proteins are added as nodes, and edges between these nodes and cluster nodes are added in a manner similar to the construction of cluster graph. We, then, use Algorithm 1 to compute edge disjoint paths from the query proteins to the clusters. Step 6 is also modified, as the edges in the cluster graph represent a set of edges, and each edge disjoint path in the cluster graph represents one or more edge disjoint paths in the interaction network; but this number cannot be higher than the number of edges that the first edge from the query node represents. We name this number as the *bandwidth* of the path. The flow along the path cannot be greater than this bandwidth. So we modify (1) as *R* ≥ 1 − ∏ _*i*=1_^*d*^ *P*_*i*_^bandwidth_*i*_^. We then use these maximum bounds to order and prune clusters for queries. Figure 5 illustrates the construction of the cluster graph. In the figure, there are four cluster nodes and one query protein represented by a black circle. The computation of a weight between two clusters is shown on the left. Two gray clusters have 7 members each, and there are three edges between their members in the original network. The weight of the edge between these two clusters is defined as the maximum weight of the three edges. The remaining edges in the cluster graph are computed in a similar manner.

### 4.1. Single Level Clustering

 We propose multiple clustering schemes to scale Net-Flow. The first one, spectral clustering, is shown to be successful where interobject similarity is defined rather than interobject distances [18]. Interaction networks inherently provide a similarity matrix, *M*, where each entry *M*(*i*, *j*) represents the probability that protein *i* and protein *j* interact. We first find an eigenvalue-decomposition of *M* and perform *k*-means clustering by using the eigenvectors with the *k* largest eigen values. The advantages of this clustering technique are that the number of clusters can be easily changed to obtain clusters of different resolutions. We applied spectral clustering to the yeast network which has 3112 proteins. The resulting clusters are, however, not satisfactory. There is a large set of nodes that seem to be almost equidistant to each other, and thus cannot be clustered into different clusters. When *k*, the number of clusters, is 20 there is a cluster with 1800 elements. When *k* is 50 there is a cluster with 818 elements. This nonuniformity of the cluster sizes reduces the efficiency of clustering.

The second technique is called the Nearest Neighbor Clustering (NNC) and is a variation of the neighbor joining algorithm [19]. Experimentally, we have seen that the yeast network contains a small number of pairs of proteins that have high probabilities of interaction, and the number of interacting pairs increases rapidly as the probability values decrease. In maximum bound calculations, the edge with the maximum probability between clusters is extremely important as it is used to assign edge weights while the other edges are ignored in cluster graph. The NNC method is designed to overcome the deficiencies of the spectral clustering: the maximum weight edge between clusters is minimized, and the number of elements in clusters is kept almost uniform. This algorithm starts clustering by using the edges with maximum weights. For each edge, if the nodes of the edge do not belong to any cluster, they are combined to create a new cluster. If only one of them belongs to a cluster, then the other is inserted into that cluster. If both are from different clusters, then their clusters are merged into a new cluster. For uniformity, there is a size constraint on the clusters. On the yeast network with a size constraint of 100, NNC is able to create 65 clusters that are not connected with high-weight edges.

### 4.2. Hierarchical Clustering

We have discussed how to design and construct a flat level of clusters, but it is well known that hierarchical clustering can improve the performance further. Hierarchical clustering enables us to optimize the bottleneck step 4 as well. In this scheme, the lowest level of clusters contains small number of proteins for which strong evidence exists that they interact. At the next level, the lowest level clusters are grouped based on the likelihood of interaction among their members. This process is carried out until the top level clusters are constructed. So, members of a top-most level cluster are expected to interact with each other with low probabilities, and members of the lowest-level cluster are almost certainly interacting with each other. Functional relationship queries can be efficiently answered by using this hierarchical clustering scheme. First, bounds on the reliability of the highest level clusters are computed. The most promising cluster that has the highest reliability bounds is expanded and bounds for its subclusters at the next level are computed. At the next step, bounds for all expanded cluster are considered and the most promising cluster is expanded again. This recursive procedure is carried on until all clusters are expanded or bounds of the unexpanded clusters guarantee that their expansion does not change the answer set. This algorithm is a variation of the nearest neighbor and range queries on spatial index structures [20].

**Figure 6:**
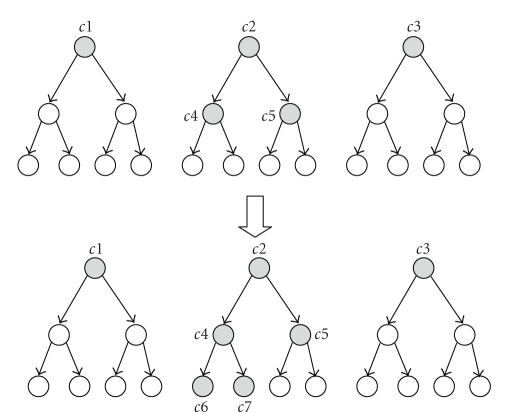
Hierarchical cluster search. First a cluster graph of the top most level is created and the maximum bound for the clusters is computed. Then the clusters that exceed the threshold are expanded further. In the figure the gray clusters represent the expanded ones. Only two of the eight leaf clusters are expanded.

This search can be configured to answer threshold queries where the proteins whose reliability is greater than some threshold are returned with high efficiency. Since in each computational step, the maximum reliability for each cluster is known, one can expand clusters using this bound. For example, if the maximum reliability bound of a cluster does not exceed the given threshold its members can be safely eliminated from the candidate set. This elimination makes the search technique faster. On top of that, this scheme can be used to answer threshold queries and adaptively change the threshold based on the query. Figure 6 illustrates querying using hierarchical clustering. In the first step a cluster graph is constructed using cluster set of *c*1, *c*2, *c*3. Maximum reliability of each cluster is computed. Let us assume that the reliability of *c*2 is the maximum and it exceeds the given threshold. Then *c*2 is expanded, and a new cluster graph is constructed with clusters *c*2, *c*3, *c*4, *c*5. On this new graph the maximum bounds of the new clusters *c*4 and *c*5 are computed. For example, if the threshold is too high so that none of the maximum bounds of these clusters exceed the threshold at this point, then the threshold is decreased. Let us assume that only the bound of *c*4 exceeds the threshold. Then, *c*4 is expanded and the new cluster set becomes *c*1, *c*3, *c*5, *c*6, *c*7. The maximum bounds of *c*6 and *c*7 are computed on the new cluster graph constructed using the new cluster set. Let us assume that the maximum bounds of both *c*6 and *c*7 exceed the threshold. Then these clusters need to be expanded, but since they are leaf level clusters their members are added to the candidate list. Then the reliability of the proteins in the candidate list is computed using the whole protein network. For the case illustrated in Figure 6, there are 8-leaf level clusters that contain all of the proteins in the network. And after cluster search it is found that only two of these clusters exceed the threshold decreasing the size of candidate set to a quarter of the size of initial set. The time spent on searching the hierarchical clustering is marginal compared to running network flow instances for the candidate proteins. In our experiments, hierarchical cluster search takes about 17 minutes compared to 10 hours spent on running network flow instances.

One can use hierarchical clustering of the network to compute approximate results with desired speedup. A search technique like the one described above will guarantee that the clusters of the proteins that are most likely to interact with the query complex members are expanded first because at each step the cluster with the maximum bound is considered. Thus, during this search an ordered list of candidates is created, where the most promising ones are at the top. An expensive network flow instance, then, can be run using the top-ranked proteins. The number of candidates that are considered determines the achieved speedup. For example, if only the top 1% of the list is checked with network flow, a speedup of 100 is achieved.

## 5. Experimental Evaluation

Many biological studies for identification of functional interactions between proteins have targeted the model organism yeast due to its small genome, extensive genetic information, and well-known biochemistry. Therefore, yeast genome is used in most of computational studies on protein networks because of the availability of extensive experimental data. The probabilistic yeast network network, ProNet [4], used in experiments was built using four experimental data sources [21–24]. The resulting probabilistic network contains 3112 genes connected by 12594 undirected edges weighted by the computed probabilities.

**Figure 7:**
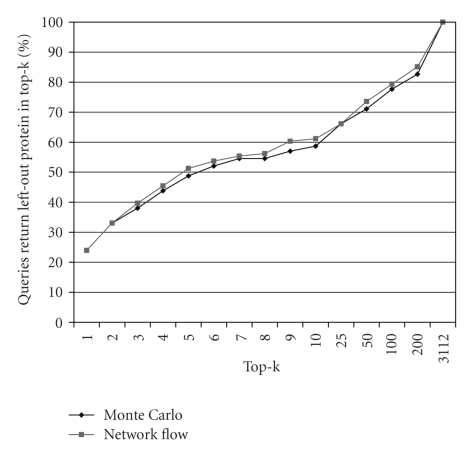
The percentage of leave-one-out queries that return the left-out protein in top-k.

**Figure 8:**
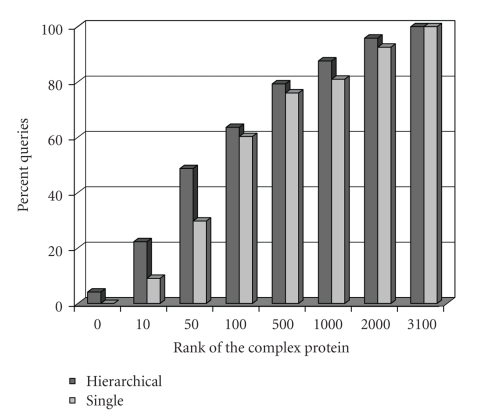
The percentage of leave-one-out queries that return the left-out protein in top-k using single-level and hierarchical clustering.

To construct membership queries, we assembled a benchmark dataset of protein complexes (direct interaction). The benchmark consists of 27 complexes from the MIPS database of yeast protein complexes (same validation dataset used in [4]). The complexes vary in size between 3 and 15 proteins. We perform leave-one-out experiments on these complexes. For each complex in the benchmark dataset, one member of a complex is left out and the remaining proteins in the complex are used as the partially known complex. We choose to define our queries as such because this guarantees that the proteins used in the query are functionally interacting, and a membership query should return proteins that functionally interact as well. Figure 7 shows the percentage of leave-one-out queries that return the left-out protein in top-k. Furthermore, Figure 8 shows that approximating the reliability values using clustering does not sacrifice accuracy. These percentages highly depend on the completeness and correctness of the underlying network. In the next section, we show that instead of using ranking queries, threshold queries provide more meaningful results.

### 5.1. Threshold Queries

**Figure 9:**
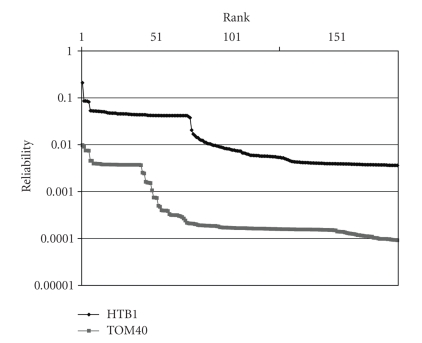
The reliability of the top-ranked results of two complex membership queries.

A common query for the network analysis techniques is to identify the relevance of the rest of the proteins in the proteome to a partially known complex or pathway. A measure of the performance uses the relevance of the top-ranked results to query proteins. Because of the diverse nature of such queries, definition of the top-ranked results is difficult. In our experiments, we have seen that the reliability of proteins in the ordered list rapidly decreases after a few top-ranked results. After some ranks, the reliability of the proteins is very close to each other and ranking one of them over another is not statistically significant. Figure 9 depicts the behavior of two complex membership queries, *HTB1* is left out from the nucleosomal protein complex and *TOM40* is left out from transport across the outer membrane complex. As can be seen from the figure there is not a common threshold or top-k elements that would be meaningful for both queries. In cases such as these, an adaptive threshold query would produce meaningful results since it considers both ranks and corresponding values. For *HTB1*, a threshold of 0.03 is suitable and for *TOM40*, a threshold of 0.003 would produce meaningful results. Since there is not a specific threshold that is suitable for all queries, a successful approach has to be adaptive to optimize the performance gain in these situations.

**Figure 10:**
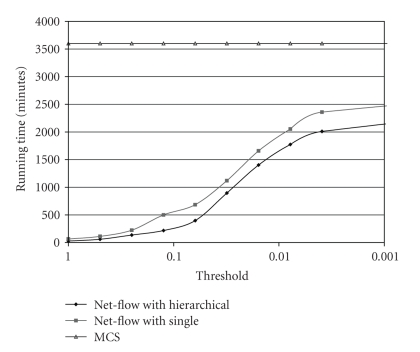
Running time of Net-Flow and MCS to find proteins whose reliability is greater than some threshold.

The flexible approach of Net-Flow is adaptive enough to answer threshold queries with maximum performance gain. In Net-Flow a maximum bound for the reliability of each cluster is computed. So, if this bound is less than the threshold, the members of this cluster can be eliminated from the candidate set without further consideration. The clusters that exceed the threshold are expanded to lower level clusters in hierarchical clustering to gain finer bounds. If the threshold defined initially is too high to return meaningful answers, it can dynamically be decreased without loss of any computation power. Figure 10 shows the running time of Net-Flow with single level and hierarchical clustering schemes. MCS is plotted with a straight line because it does not adapt to the threshold queries. Net-Flow is very adaptive with both of the clustering schemes. Especially for the large threshold values, speedups of two order of magnitudes are achieved. Even for small threshold values when the answer set size is larger it runs much faster than MCS.

**Figure 11:**
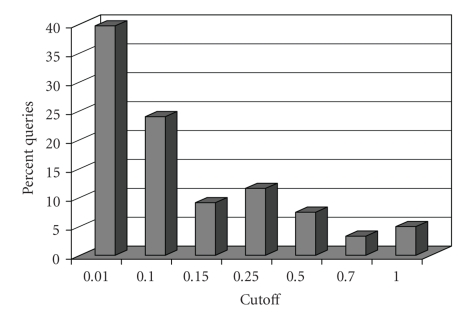
Histogram of the reliabilities of the left out protein in leave-one-out complex experiments for Net-Flow.

To ensure that the threshold queries can recognize interacting proteins, we analyzed the reliability assigned to the left-out proteins in leave-one-out experiments. Figure 11 displays the results for the complex queries. As seen in the previous experiments these reliabilities show a large variation. Small numbers of left-out proteins possess high degrees of reliability to the rest of the complex members while high numbers of the proteins possess low degrees of reliability. This shows that the amount of noise is too high in the network and in many cases there is not sufficient amount of experimental data to support the functional interaction relations among complex members. However more than 60% of the queries can be found with a threshold of 0.01. For this threshold Net-Flow has a speedup of nearly 4.

**Figure 12:**
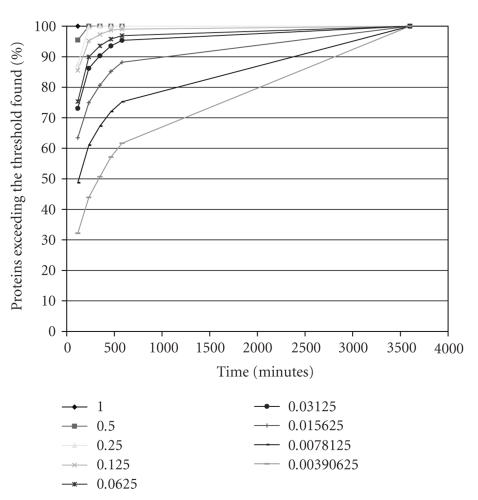
The percentage of elements with reliability greater than some cutoff successfully found by using different levels of speedup using hierarchical clustering.

On top of answering the threshold queries to find all the proteins whose reliability exceed some threshold, Net-Flow can produce approximate results for a given speedup as well. Figure 12 depicts the accuracy of Net-Flow with varying speedups for a set of thresholds. As can be seen Net-Flow scales best for high thresholds. Even when the running time is 10 times faster Net-Flow is able to give accurate approximate answers. For thresholds higher than 0.0625, it is able to give answer queries with more than 90% accuracy. The accuracy naturally drops as the threshold decreases because the result set gets larger and the noise increases. High accuracy for large thresholds compared to that of smaller thresholds shows that Net-Flow finds the proteins with high reliability first.

## 6. Discussion

In this paper we analyzed two classes of techniques for the analysis of protein interaction networks. We showed that methods that do not penalize high degree nodes have advantages over the methods that penalize high degree nodes. We proposed a new analysis technique based on network flow. This technique computes bounds on the reliability of the connections between two nodes. These bounds are theoretically proven unlike previously proposed ones. We tested this new network flow-based technique, Net-Flow, using leave-one-out complex membership queries. We also integrated a hierarchical clustering component of Net-Flow allowing to answer threshold queries efficiently. We have shown that Net-Flow can run 10 times faster for meaningful threshold queries than a competing technique based on Monte Carlo simulation.

## Figures and Tables

**Algorithm 1:**
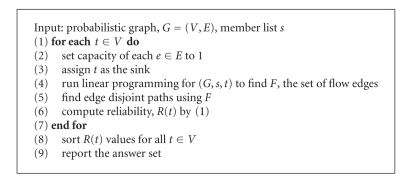
General flow algorithm.
